# Does Abiotic Host Stress Favour Dothideomycete-Induced Disease Development?

**DOI:** 10.3390/plants11121615

**Published:** 2022-06-20

**Authors:** Laura Röhrig, Francois Dussart

**Affiliations:** 1 Department of Agriculture, Horticulture and Engineering Science, Scotland’s Rural College (SRUC), Edinburgh EH9 3JG, UK; laura.roehrig@sruc.ac.uk; 2 Institute of Molecular Plant Sciences, School of Biological Sciences, University of Edinburgh, Edinburgh EH9 3BF, UK

**Keywords:** Dothideomycetes, plant pathogenic fungi, host stress, abiotic stress

## Abstract

Dothideomycetes represent one of the largest and diverse class of fungi. This class exhibits a wide diversity of lifestyles, including endophytic, saprophytic, pathogenic and parasitic organisms. Plant pathogenic fungi are particularly common within the Dothideomycetes and are primarily found within the orders of Pleosporales, Botryosphaeriales and Capnodiales. As many Dothideomycetes can infect crops used as staple foods around the world, such as rice, wheat, maize or banana, this class of fungi is highly relevant to food security. In the context of climate change, food security faces unprecedented pressure. The benefits of a more plant-based diet to both health and climate have long been established, therefore the demand for crop production is expected to increase. Further adding pressure on food security, both the prevalence of diseases caused by fungi and the yield losses associated with abiotic stresses on crops are forecast to increase in all climate change scenarios. Furthermore, abiotic stresses can greatly influence the outcome of the host-pathogen interaction. This review focuses on the impact of abiotic stresses on the host in the development of diseases caused by Dothideomycete fungi.

## 1. Introduction

The sessile nature of most land plants implies that these organisms cannot escape changes in their environment. Plants are constantly subjected to various stresses throughout their life, be it biotic stresses that are due to pathogen or herbivory attacks, or abiotic stresses that result from harmful growing conditions. Through evolution, plants have acquired a plethora of mechanisms that allow them to withstand or even thrive in these conditions. However, with climate change, changes in environmental conditions occur more frequently and more suddenly than ever before, putting these adaptation mechanisms under pressure. All climate change models forecast that plants are likely to face an increased pressure in the future. Indeed, plant pathogens, particularly fungi and oomycetes, are forecast to move towards the poles in a warming climate, exposing major crops to new pathogens [1,2]. Climate change models also predict that plants will be subjected to increased abiotic stresses. Abiotic stresses, which can be defined as the negative influence of non-living factors (also known as stressors) on plant development and physiology, result from changes in the environment due to the variation of the stressors beyond their normal range. These stressors include extreme temperature, increase in UV radiation and CO_2_ levels, changes in rainfall patterns resulting in drought and flooding, changes in soil chemical composition associated with leaching of nutrients, increased salinity and changing pH and increased frequency of extreme weather events occurring, such as flash floods and storms [3].

Abiotic stress is one of the main contributors to yield losses worldwide as it accounts for up to 50% of major crop yield losses [4] and is an important threat to food security in the context of climate change. Currently, almost half of arable land is estimated to be at risk of drought [5], and salinisation of arable land further increases pressure on yield and reduces the acreage available to grow crops [6]. Furthermore, anthropogenic activities, including the application of agrochemical products, such as fertilisers and pesticides, have led to an increase in heavy metals concentration in the soil, particularly cadmium (Cd), lead (Pb), arsenic (As) and chromium (Cr) [7]. In addition to reducing yield through abiotic stresses, these heavy metals can also be found in the harvested products, raising concerns for human and animal health [8]. Therefore, new agronomic management strategies and increasing crop tolerance to abiotic stresses are high on the agenda of most crop improvement programmes [9]. Recent research suggests that supplying crops with plant primary and secondary metabolites, such as polyols, can help increase tolerance to various stresses, including drought, salinity, temperature, and heavy metals [5]. Similarly, a study carried out by Redman et al. [10] suggests that colonisation of rice crops with endophytic fungi increases crop fitness and confers tolerance to both drought and salinity stress. However, in the field, plants face multiple stresses at the same time, which poses a challenge to the development of stress-tolerant plants [11].

In addition to direct yield losses, abiotic stress can also lead to indirect losses as a result of increased susceptibility to pathogens. Salt stress in cucumber has been associated with increased susceptibility to the bacterial pathogen *Pseudomonas syringae* pv. *lachrymans* [12]. Vaughan et al. [13] showed that growing maize plants under either drought or elevated CO_2_ stress resulted in higher infection by *Fusarium verticillioides* compared to plants grown without abiotic stress. Interestingly, plants grown under a combination of both salt and elevated CO_2_ stresses showed even higher disease susceptibility compared to plants grown under a single stress. Similarly, Sewelam et al. [14] showed that *Arabidopsis thaliana* plants subjected to combined heat and osmotic stress exhibited increased susceptibility to fungal and bacterial pathogens. The existence of a cross talk between the signalling response to abiotic and biotic stress is thought to underlie this relationship, as the plant response to abiotic stresses involves a multitude of phytohormones, including salicylic acid (SA), jasmonic acid (JA) and abscisic acid (ABA), which are key hormones in the response to biotic stresses [15,16]. Huot et al. [17] showed that elevated temperature partially suppresses SA signalling in *A. thaliana* and results in increased susceptibility to *P. syringae*. Vaughan et al. [18] also showed that increased CO_2_ levels results in impaired SA and JA pathway induction in maize plants. Furthermore, Sewelam et al. [14] showed that under the influence of combined abiotic stresses, plants challenged by pathogens exhibited reduced expression of defence-related genes, resulting in increased susceptibility to diseases. In addition to partially suppressing inducible plant defences, abiotic stresses also have an influence on constitutive defences in plants, resulting in increased susceptibility to pathogens. Maize plants subjected to a combination of drought and high CO_2_ levels exhibit lower levels of 2,4-dihydroxy-7-methoxy-1,4-benzoxazin-3-one (DIMBOA) glucopyranose, a well-known reserve form of the benzoxazinoid DIMBOA involved in constitutive plant defences against a broad spectrum of pathogens [13].

The class of Dothideomycete is the largest class of Ascomycota and is arguably one of the most relevant to food production, as it contains some of the most important plant pathogenic fungi [19]. Several Dothideomycetes affect crops used as staple food around the world. To list only a few, *Zymoseptoria tritici* (teleomorph *Mycosphaerella graminicola*) and *Dreschlera tritici-repentis* (teleomorph *Pyrenophora tritici-repentis*), the causative agents of Septoria tritici blotch and tan spot, respectively, are major pathogens of wheat worldwide [20,21], and black Sigatoka disease caused by *Pseudocercospora fijiensis* (teleomorph *Mycosphaerella fijiensis*) remains a threat to banana production in many countries [22,23]. Given that all climate change models forecast an increase in abiotic stress to crops around the world and that these stresses have an impact on plant-pathogen interactions, it is important to understand how abiotic stresses can affect disease prevalence and severity. This review focuses on the effect of abiotic stresses on the host in the interactions between the plant and pathogenic Dothideomycete fungi.

## 2. Overview of the Abiotic Stress Response in Plants

The survival of plants depends on their ability to perceive and respond to stress factors they encounter as sessile organisms. Plants have evolved complex mechanisms to respond to an ever-changing environment [24]. The abiotic stress response can be divided into a short-term response, which occurs within hours of the stress, or a long-term response that can be mounted several days after the stress occurred. Although each stress elicits a unique response from the plant (these will be discussed in more detail in each section), some elements of the response are common to many different stresses. These include, for instance, calcium (Ca^2+^ ) signalling, activation of kinase cascades, metabolite sensing, phytohormone balance and the production of reactive oxygen species (ROS) [25,26]. Ca^2+^ signalling is considered as a universal secondary messenger for the primary stress response that is elicited into the cytosol upon both biotic and abiotic stresses [27]. This in turn activates other calcium binding proteins, such as calcineurin-B-like proteins (CBLs), calmodulin (CaM) and CaM-like proteins (CMLs) [28,29]. In addition, calcium-dependent protein kinases (CDPKs), histidine kinases (HKs), receptor-like kinases (RLKs) and mitogen-activated protein kinases (MAPKs) play an important role in the transduction of the signal by the stress signalling network, resulting in transcriptional reprogramming to alleviate the stress [30,31,32]. 

One of the first responses to abiotic stress is the production of phytohormones that act as signalling molecules, resulting in the expression of stress-related genes [33,34]. For example, ABA is known to mediate stomatal closure during drought stress conditions by regulating plasma membrane transporters via phosphorylation by downstream CDPKs [35]. Phytohormones such as cytokinins, gibberellins (GA), ABA, ethylene (ET), SA, brassinosteroids (BR) and JA are major plant defence regulators [36,37]. The different roles of phytohormones involved in plant defence, growth and development have been previously reviewed in great detail elsewhere [26,36,38,39,40] and will not be discussed further here. 

During abiotic stress, two major sources of ROS are metabolic ROS that are produced from disruptions in metabolic activity; and signalling ROS that result from abiotic stress-signal transduction [41]. ROS are reduced or activated forms of atmospheric oxygen, such as hydrogen peroxide, superoxide, hydroxyl radical and singlet oxygen [42], that are primarily produced in the chloroplasts, mitochondria, peroxisomes and the apoplast during the abiotic stress response [38]. Each cellular compartment produces and controls its own ROS homeostasis, and thus, different ROS levels in different compartments create a distinct ROS signature [38]. In the apoplast, for example, ROS are mediated by membrane bound NADPH-oxidases, including respiratory burst oxidase homolog D (RBOHD) [30,38,43]. To minimise the oxidative damage to DNA, lipids and proteins caused by ROS, plants can produce antioxidants, including ascorbic acid and glutathione, as well as ROS-scavenging enzymes, such as superoxide dismutase (SOD), ascorbate peroxidase (APX), catalase (CAT) and glutathione (GSH) peroxidase [41,44]. For example, in chloroplasts, ROS can be alleviated by ROS-scavengers such as iron- and copper-zinc-SODs as well as APXs and glutathione [38,44]. However, it is important to note that combined stresses elicit complex responses that cannot be forecast based on the response to each individual stress, but rather represent a unique stress response [45,46]. 

## 3. Temperature Stress Affects Dothideomycete-Induced Disease Development

**Figure 1:**
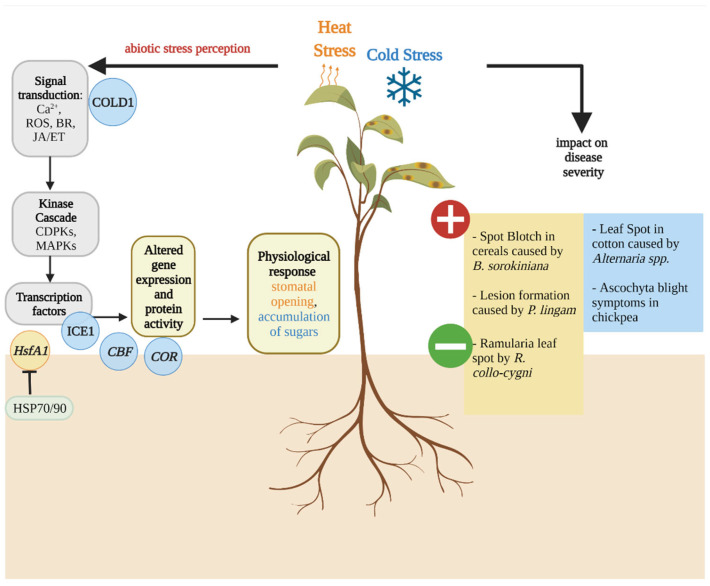
Overview of host temperature stress signalling pathways and the impact of temperature stress on Dothideomycete-induced disease severity. Figure 1 highlights the signalling pathways (**left**) and effects of cold (blue) and heat stress (orange) on the severity of several Dothideomycete-induced diseases (**right**). The cold response cascade presented is shared between *A. thaliana* and rice. The membrane protein chilling tolerance divergence 1 (COLD1) triggers the release of calcium ions (Ca^2+^) upon the perception of freezing and chilling stress. Mitogen-activated protein kinase (MAPK) mediates this signalling via the expression of transcription factors such as inducer of C-repeat binding factor (CBF) expression 1 (ICE1), which in turn activates the expression of *cold responsive* (*COR*) genes via transcriptional activators such as CBF. The ICE1-CBF-COR pathway is a key response pathway to cold stress. The change of membrane fluidity upon heat stress is proposed to induce a signalling cascade mediated by Ca^2+^. The heat shock proteins HSP70/90 repress the activity of the heat stress transcription factor HsfA1, a master regulator of thermotolerance in the absence of heat stress. Upon heat stress, this regulator is activated and targets downstream transcription factors, which in turn modulate the synthesis of chaperones and enzymes involved in degradation of unfolded proteins and scavenging of reactive oxygen species (ROS).

Along with water, nutrients and light, an optimum temperature is required for successful plant growth. The optimal temperature differs depending on the plant species, the age and duration of exposure to the temperature; however, every plant can be subjected to temperature stress either as heat stress, when temperature increases, or cold and freezing stress, when temperature drops far below the plant’s optimal temperature range [47]. The membrane fluidity can either decrease or increase upon cold and heat stress, respectively, which activates the Ca^2+^ -mediated signalling cascade (Figure 1) [27]. Within the 250 calcium sensor proteins estimated to be present in *Arabidopsis thaliana* [48], cyclic-nucleotide gated Ca^2+^ channel 6 (CNGC6) is known to mediate heat-induced Ca^2+^ influx that stimulates the expression of *heat shock protein* (*HSP*) genes leading to increased heat tolerance [49]. The heat shock proteins HSP70/90 can repress the activity of heat stress transcription factors (Hsf) such as HsfA1, a master regulator of thermotolerance in the absence of heat stress. HsfA1 activates the expression of heat stress-responsive genes such as *dehydration-responsive element binding protein 2A* (*DREB2A*), which is an important regulator of thermotolerance in *A. thaliana* [50]. In *A. thaliana*, the C-repeat/DREB1 (CBF) pathway has been described to play an important role in cold acclimation [51]. The expression of *cold-responsive* (*COR*) genes is mediated by mitogen-activated protein kinases (MAPKs) that activate the transcription factor inducer of CBF expression 1 (ICE1) via the transcriptional activators *CBFs* [27]. Phytohormones such as BR, ET and JA interact with the ICE-CBF-*COR* pathway by either activating or repressing the expression of genes in this pathway [27].

### 3.1. Effect of Host Heat Stress on Dothideomycete-Induced Diseases

At elevated temperatures, typically above 30 °C, plant growth, development and seed germination are among the processes that are negatively impacted the most [52]. At the cellular and molecular level, elevated temperatures affect the stability of proteins, RNA and cytoskeletal structures and initiate metabolic imbalances [43,52,53]. Photosynthesis is a highly temperature-sensitive cell process that declines by 40% when plants are subjected to temperatures above 30 to 35 °C [47,53,54]. Heat stress during the reproductive development of cereal crops leads to a significant reduction in floret fertility, resulting in reduced seed number [55]. Post-anthesis heat stress also affects grain filling by hindering translocation of photosynthates to the grain, which in turn affects starch synthesis, leading to a reduction in thousand grain weight [55,56].

With regards to Dothideomycete-induced diseases, heat stress can also affect host resistance to pathogens [57]. The hemibiotrophic fungus *Bipolaris sorokiniana* (teleomorph *Cochliobolus sativus*) is the causative agent of spot blotch in cereals, a disease particularly prevalent in warm, humid climates around the world [58]. In wheat crops in India, it was estimated that the average yield loss due to spot blotch ranges between 15% to 25% [59]. The disease also impacts grain quality by reducing the 1000-kernel weight (TKW) [60]. Severe disease outbreaks have been observed when high temperatures and high relative humidity coincide with the late post-anthesis growth stage (GS) of wheat (GS 69) [58,61]. In India, heat stress associated with constant temperatures above 26 °C favoured the development of spot blotch in wheat, and similar observations from Nepal and Bangladesh indicated that especially the combination of terminal heat stress and spot blotch were the main causes for the loss of crop productivity [62,63]. Sharma et al. [64] examined a six-year dataset at 11 different sites within the Eastern Gangetic Plains to assess the relationship between disease severity, TKW and temperature in spring wheat. The study confirmed a trend towards increasing night-time temperature and increased spot blotch severity, decreasing TKW and yield over the six-year period [64]. The authors postulated that the elevated temperatures during the night may affect plant physiology, as an increase in dark respiration would lead to premature leaf senescence. Similar observations were made in a study by Mercado-Vergnes et al. [65], which indicated a major shift in susceptibility to *B. sorokiniana* between anthesis (GS 61) and late milk grain stages (GS 77) in five wheat genotypes. Taken together, these results suggest that physiological changes in the host could also be related to the onset of leaf senescence with changes in source-sink metabolism, and thus, affect disease susceptibility [65]. However, it should be noted that in these studies, increased disease levels cannot be solely attributed to heat-induced host stress, as temperature could also alter *B. sorokiniana* growth. Further studies are required to fully understand the mechanisms underlying these observations.

Similarly, plant ontogenesis as well as abiotic stress factors such as elevated temperatures were also observed to play a role in the development of the barley disease, Ramularia leaf spot (RLS), caused by *Ramularia collo-cygni* (teleomorph unknown) [66,67]. However, for RLS development a combination of abiotic factors as well as the host GS appear to be crucial [66,68]. In the Czech Republic, lower disease symptoms were observed when lower rainfall and higher temperatures occurred post heading (GS 59) [69]. Similar observations were made in Germany, where lower RLS severity was recorded in the warmer and drier years of 2017 and 2019 [70]. Hoheneder et al. [70] suggested that cellular responses to heat and drought, such as hormonal imbalances caused by ROS, osmotic adjustments and accumulation of antioxidants could inhibit the development of *R. collo-cygni*, and therefore, decrease RLS severity. 

Analysing bulk samples of winter wheat grain from 1844 to 2003 in the UK revealed a negative correlation between high temperatures and *Parastagnorospora nodorum* DNA in grains. *P. nodorum* (teleomorph *Phaeosphaeria nodorum*) is the causative agent of Septoria nodorum blotch (SNB) and glume blotch in wheat and other cereals. [71,72]. SNB reduces the photosynthetic area of upper leaves, leading to yield losses of up to 30%, and glume blotch is known to affect grain quality [73,74]. It was reported that both seed germination and seedling emergence were reduced after glume blotch seed infection [75]. *P. nodorum* often occurs alongside two other necrotrophic Dothideomycetes on wheat, *Z. tritici* and *D. tritici-repentis*, responsible for Septoria tritici blotch (STB) and tan spot, respectively. Before the Green Revolution in the 1980s, *P. nodorum* was the major pathogen of the wheat blotch complex in Europe; however, in northern Europe, STB has increasingly become more prevalent in wheat crops. This shift in disease prevalence was mainly attributed to climatic changes, including increased temperature, altered rainfall patterns and increased air pollution [72,73]. Whether this change in STB prevalence in northern Europe is due to a better adaption of *Z. tritici* to fungicide programmes, increased *Z. tritici* host susceptibility or climatic conditions favouring disease progression is still unclear [73]. Further studies are required to fully understand the mechanisms underlying these observations. 

A recent study highlighted that temperature could also play a role in increasing lesion formation of *Phoma lingam* (teleomorph *Leptosphaeria maculans*) in spring and winter oilseed rape (*Brassica napus*), in which it causes blackleg or Phoma stem canker [76]. The disease causes major economic losses in oilseed rape growing areas of Europe, Australia and Canada. Yang et al. [76] studied the effect of temperature on the hypersensitivity response (HR) to *P. lingam* in two oilseed rape genotypes and found that lesion formation was increased in plants grown under elevated temperatures in controlled environment experiments. The expression levels of two plant defence marker genes, *PATHOGENESIS–RELATED* (*PR*) *1* and *PR 2* and the temperature-sensitive gene *BONZAI 1* (*BON1*)*,* were increased in response to elevated temperature in infected *B. napus* [76]. The observed increase in lesion size in both investigated genotypes was hypothesised to be the result of the mitigation of the HR on the suppression of fungal growth by the increase in temperature. Similar observations were made in controlled environment experiments in which the R gene *Rlm6*-mediated resistance to *P. lingam* appeared to be sensitive to temperature [77]. Development of large lesions were observed on resistant cultivars at 25 °C, but not at temperatures below 20 °C [77]. Huang et al. [77] suggested that if oilseed rape cultivars carry such temperature-dependent R genes, it could help to explain why Phoma stem canker is more severe in Western Australia where temperatures reach a maximum of 30 °C compared to the European oilseed rape season where it reaches only 21 °C [78]. These studies emphasise the potential for greater risks of Phoma stem canker epidemics in Europe where temperatures are predicted to increase [77,79]. This also highlights the importance of further investigating the impact of abiotic stresses, such as high temperatures, on the host response, and to understand the mechanisms that regulate the interaction between host, environment and pathogens.

### 3.2. Effect of Host Cold Stress on Dothideomycete-Induced Diseases

In addition to heat stress, low temperature stress is one of the most common abiotic stresses that plants are exposed to. Low temperature stress is divided into chilling stress, when temperatures remain above 0 °C, and freezing stress, when temperatures drop below 0 °C. Although most temperate plants can tolerate freezing temperatures through cold acclimation, tropical and subtropical plants lack this ability and are sensitive to chilling stress [80,81]. Such plants include major crops such as rice, corn, soybean, banana, cotton and tomato. Cold stress causes cell death primarily induced by cellular dehydration, formation of ice in cell walls and damage to cell membranes, resulting in chlorosis and necrosis [82]. The process of cold acclimation is associated with changes in the composition of lipids, increases in sugar and protein content and changes in hormone levels in the plant cell [83]. Freezing tolerance is linked to acclimation processes, which include cellular osmotic stabilisation, antioxidant production and photosynthesis adjustments [84]. 

With regards to Dothideomycete-induced diseases, chilling can predispose crops to pathogen infection [85,86]. Observations from China reported that chilling stress acted as an essential predisposing factor for cotton leaf spot caused by *Alternaria alternata* (teleomorph unknown) [85]. *A. alternata* and *A. macrospora* are the two main causative agents of leaf spot in cotton and can cause major economic damage [87]. Cotton leaf spot is responsible for yield losses of 37% in India, the biggest producer of cotton in the world [85,88]. *A. alternata* was linked to cotton leaf senescence, both under field and controlled conditions. In the field, enhanced premature leaf senescence was observed together with leaf spot lesions after periods of temperatures as low as 8.4 °C. The relationship between senescence, chilling and lesion appearance was further investigated under controlled conditions and showed that cotton leaves exposed to chilling stress prior to *A. alternata* inoculations exhibited premature leaf senescence contributing to yield loss. Moreover, cotton leaves exposed to chilling stress prior to *A. alternata* inoculations also exhibited increased leaf spot symptoms compared to plants grown under normal conditions [85]. Whether chilling-induced physiological impairment caused a leakage of ions and nutrients that favoured colonisation by *A. alternata* or a reduction of tissue resistance to *A. alternata* due to chilling increased the severity of cotton leaf spot is not fully understood yet [85]. 

Similar observations were found for winter sown chickpea cultivars in Northwest Asia and North Africa [86]. Kemal et al. [86] reported that chilling and freezing temperatures can predispose chickpea to severe Ascochyta blight. Ascochyta blights represent the most important threat in cool season food legumes such as chickpeas, peas, lentils and faba beans [89]. Chickpea, the second most important legume worldwide by production, is particularly susceptible to Ascochyta blight caused by *Ascochyta rabiei* (teleomorph *Didymella rabiei*) [90,91]. At present, the disease occurs in more than 30 countries in Northwest Asia, North Africa, southern Europe, Canada and Australia [91]. Kemal et al. [86] investigated the effects of temperature on pathogen aggressiveness and host resistance under controlled conditions using five chickpea cultivars and four *A. rabiei* isolates. The study indicated that disease resistance in chickpea is not temperature dependent in the investigated genotypes and does not enhance the aggressiveness of the studied isolates. However, the authors found that chilling various chickpea cultivars differing in their resistance to Ascochyta blight resulted in increased disease development. Further supporting the role of chilling stress on disease development, fungal isolates associated with low aggressiveness exhibited increased disease severity in plants exposed to chilling stress prior to inoculation compared to plants grown without chilling stress [86]. These results indicate that chilling stress in chickpea favours the development of Ascochyta blight. However, to our knowledge, no studies have investigated the mechanisms underlying these observations.

## 4. Water Stress in the Host Affects Pathogenic Dothideomycete-Plant Interactions 

**Figure 2:**
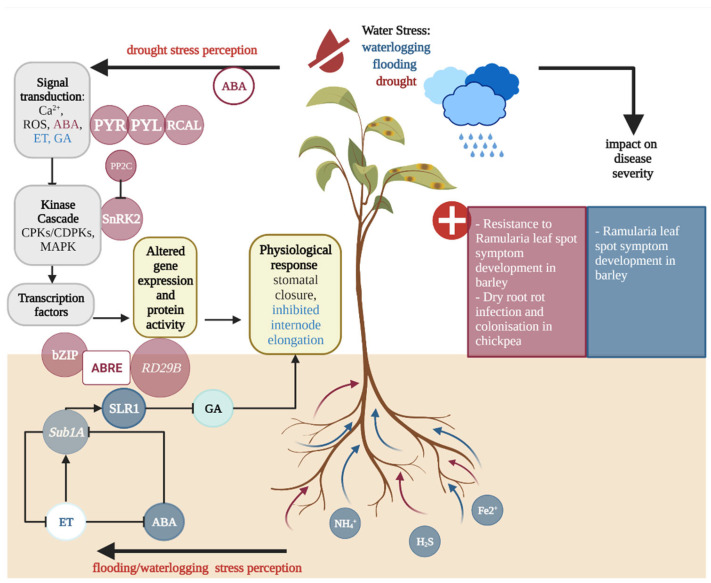
Overview of the impact of host water stress signalling pathways and the impact of water stress on Dothideomycete-induced disease severity. Figure 2 highlights the signalling pathways (**left**) and the effects of drought (dark red) and waterlogging and flooding (dark blue) on Dothideomycete-induced disease severity (**right**). Upon drought stress, calcium ions (Ca^2+^) and abscisic acid (ABA) accumulate. The signalling cascade is initiated by ABA receptors such as the pyrabacting resistance (PYR)/PYR1-LIKE (PYL)/regulatory components of ABA (RCAL) proteins binding to ABA. In the presence of ABA, the PYR/PYL/RCAL complex interacts with a group of protein phosphatase 2Cs (PPC2s), which represses the activity of sucrose nonfermenting 1 (SNF1) related protein kinase 2 (SnRK2) and downstream ABA signalling. The activation of kinases such as SnRK2 leads to the activation of transcriptional factors that control drought stress responsive genes and modulates plasma membrane proteins in guard cells, resulting in stomatal closure. SnRK2 is an upstream activator of bZIP transcription factors with known ABA-responsive element binding factors (ABFs or AREBs), which play key roles in binding to cis-elements of promotor regions of ABA-responsive genes, such as the *responsive to dehydration* (*RD29B*) gene in *A. thaliana.* Similarly, waterlogging stress induces ABA synthesis in the root system, also leading to stomatal closure. In rice, it was shown that the expression of *Submergence-1A* (*Sub1A*) is induced during flooding by low levels of ethylene (ET), leading to an increase of the expression of slender rice 1 (SLR1), which is a known gibberellin suppressor that inhibits internode elongation during the short-term waterlogging response.

Globally, altered precipitation patterns are a major consequence of climate change. Current models show that annual precipitation is predicted to increase in Canada, Northeast USA, East Africa and northern, East, South and Southeast Asia as well as in North and Central Europe. In contrast, annual precipitation is likely to decrease in Central America, Southwest USA, the Mediterranean Basin, and Central Asia [92]. These changes in precipitation are likely to result in increased abiotic stresses on plants. The abiotic stress response upon drought and waterlogging is regulated by signalling pathways involving the interaction of several phytohormones (Figure 2). Under flooding stress, ABA, GA and ET orchestrate the acclimation response [93]. The *Submergence-1* (*Sub1*) locus encodes several ethylene responsive factor (ERF) DNA-binding proteins that are induced during flooding. The long-term flooding response involves the degradation of ABA by ABA-inactivating enzymes and an accumulation of the antagonist GA in subaqueous internodes, leading to stem elongation [94]. The drought stress response characterised in *A. thaliana* is initiated by ABA receptors such as pyrabacting resistance (PYR)/PYR1-LIKE (PYL)/regulatory components of ABA (RCAL) that interact with a group of protein phosphatase 2Cs (PPC2s) [95]. Downstream of ABA signalling, the activation of sucrose nonfermenting 1 (SNF1) related protein kinase 2 (SnRK2) is involved in the regulation of drought stress-responsive genes and modulates cell turgor in guard cells [27,95,96].

### 4.1. Effect of Waterlogging and Flooding Stress in the Host on Dothideomycete-Induced Disease Development

According to climate change models, altered predictions are likely to occur as extreme precipitation events rather than equally distributed falls throughout the year [97,98]. This would lead to longer waterlogging periods in winter and spring, and more extreme short-term flooding in summer [98]. It is estimated that currently one-third of the global irrigated area is at some point exposed to waterlogging, resulting in yield losses ranging from 15% to 80% [99]. Waterlogging impedes gas exchange in the rhizosphere, leading to hypoxia and anoxia, which affects the soil physicochemical properties and negatively impacts the soil microbiome [47,100]. Furthermore, the dissolution of carbonate and bicarbonate early during waterlogging may lead to an increase in soil pH [101,102]. Waterlogging is also associated with a decreased abundance of oxidised nutrients, such as nitrate (NO_3_^−^), sulfate (SO_4_^2−^) and ferric ion (Fe^3+^), and an elevated abundance of reduced compounds, such as ammonium nitrate (NH_4_^+^), hydrogen sulfide (H_2_S) and ferrous ion (Fe^2+^) [103]. Hypoxia leads to an increase of the redox potential between waterlogged soil and plants, and consequently, to the accumulation of ROS. This in turn is required for intra- and intercellular signalling; however, high ROS concentrations can damage membrane lipids, pigments, proteins, and nucleic acid [38,100]. Plants have evolved several mechanisms to mitigate the consequences of waterlogging stress. These have been extensively reviewed by Tewari et al. [100] and include: 1) modification of the plant metabolism toward anaerobic, glycolytic and fermentative metabolism; 2) upregulation of the antioxidant defence system to detoxify ROS; 3) expression of heat shock proteins in response to anoxia; 4) accumulation of osmolytes such as sugars and amino acids in response to osmotic stress; and 5) plant growth regulation by phytohormones, such as 1-aminocyclopropane-1-carboxylic acid (ACC) and ET [100,104,105]. 

With regards to Dothideomycete-induced diseases, waterlogging has been shown to alter the relationship between hosts and pathogens. Like other Mycosphaerellaceae, *R. collo-cygni* grows asymptomatically in its barley host for most of the growing season, which led to its classification as an endophyte that exhibits necrotrophic growth when symptoms occur, typically post-anthesis [67]. However, in some years the fungus remains in the endophytic stage without producing any disease symptoms [106]. This led to the hypothesis that environmental factors may play a role in the transition from endophytic to necrotrophic growth of *R. collo-cygni* [107]. Anecdotal reports from barley growers suggested that in years when plants are subjected to waterlogging in the field, RLS symptoms appeared earlier in the growing season compared to years without waterlogging. These observations were supported by experiments carried out under controlled conditions, showing that spring barley seedlings subjected to waterlogging stress prior to inoculation with *R. collo-cygni* showed significantly higher RLS symptom levels compared to plants that were not subjected to this stress. Taken together, these results suggest that changes in the host associated with waterlogging stress affect the relationship between *R. collo-cygni* and its host, resulting in increased susceptibility to RLS [108]. However, to the best of our knowledge, the exact mechanism underlying this increased disease susceptibility is currently unknown.

### 4.2. Drought Stress Affects the Development of Dothideomycete-Induced Diseases

Drought stress represents a major threat to agricultural productivity, particularly as the arable area under drought and aridity stress is forecast to increase as a result of climate change [109]. Drought stress can lead to up to 50% yield loss by adversely impacting photosynthesis, thereby decreasing plant growth and productivity [110]. The adverse effects of drought stress are attributed to hyperosmotic stress [111]. In response to reduced water uptake, stomatal closure is induced, which leads to a reduction in photosynthates due to limited CO_2_ diffusion and inhibition of both ribulose-1,5-bisphosphate carboxylase/oxygenase (Rubisco) and ATP synthesis [112]. Limited CO_2_ fixation capacity due to osmotic stress can lead to excess excitation energy that generates ROS within the chloroplast, resulting in an imbalance of redox homeostasis [112,113]. The accumulation of ROS in response to drought stress leads to lipid peroxidation in the membranes of the chloroplast and protein degradation, which appears to play a role in premature leaf senescence in plants [110,114]. 

With regards to Dothideomycete-induced diseases, drought is known to alter host-pathogen interactions. For instance, drought alters resistance to RLS in barley crops. Hoheneder et al. [70] showed that plants grown under long-lasting drought periods in the field exhibited increased resistance to RLS and lower fungal DNA levels compared to plants grown in irrigated plots [70]. The authors hypothesised that drought stress-induced leaf senescence could inhibit the formation of RLS symptoms [70]. McGrann et al. [68] showed that in the absence of stress, the overexpression of the barley *stress-responsive NAC* transcription factor *(HvSNAC1*) can reduce both the symptoms of RLS and fungal DNA under controlled environment conditions. *SNAC1* is involved in the regulation of responses to salt and drought tolerance in barley (*HvSNAC1*) and rice crops (*OsSNAC1*) [115]. *OsSNAC1* alters the expression of stress-responsive genes and targets genes that control stomatal closure as well as ROS homeostasis [115,116]. However, McGrann et al. [68] also showed that the overexpression of *SNAC1* did not impact the regulation of ROS scavengers such as ascorbate peroxidase (APX1/APX2), which suggests that the partial resistance to RLS mediated by the overexpression of *SNAC1* does not result from an enhanced tolerance to ROS-related damage [68]. The authors proposed that resistance to *R. collo-cygni* and reduced RLS symptoms are mediated by mechanisms regulated by *HvSNAC1* that are involved in tolerance to abiotic stress and inhibition of senescence [68]. Altogether, the results suggest that drought stress might activate transcription factors, such as *SNAC1*, which lead to an increased tolerance to RLS, and that the observed reduced fungal biomass under drought conditions in the field did not result from unfavourable growing conditions for the fungus. 

In contrast, drought appears to play an important role in increasing the severity and incidence of dry root rot (DRR) in chickpea [117]. The soil-borne fungus, *Macrophomina phaseolina* (teleomorph unknown) is the causative agent for DRR and can infect over 500 plant species worldwide, including some important crops such as soybean, chickpea, maize, sorghum and oilseed rape [118,119]. *M. phaseolina* is also responsible for stem canker, seedling blight, charcoal rot, wilt, leaf blight, stem blight and pre-emergence and post-emergence damping-off [120]. Many studies mention that, especially under high temperatures and soil moisture below 60%, this fungus can cause substantial yield losses [117,118,121,122]. Although drought is known to play an important role in the development of *M. phaseolina*, as dry soil favours microsclerotia survival, drought-induced host stress also plays a role in disease development [121,123,124]. Irulappan et al. [117] recently showed that under drought stress, both disease severity and *M. phaseolina* colonisation were higher than in irrigated chickpea plants. These observations were attributed to weakening of the endodermal barrier and an impaired defence response from the host. Using an RNA-seq approach, the authors showed a downregulation of genes associated with endodermal cell layer formation and JA/ET hormone regulation in response to combined drought and *M. phaseolina* stress [117]. Under pathogen stress only, the expression of several defence-related genes, such as that encoding for the jasmonate ZIM domain (JAZ) proteins, was upregulated 3 days after sowing [117]. However, if chickpea plants were exposed to drought conditions prior to inoculation, these genes were downregulated 3 days after sowing [118]. The authors hypothesised that, based on the reduced transcript levels of several defence genes during biotic and drought stress, the immune response appears to be suppressed, resulting in increased disease severity [117]. 

## 5. Salt Stress Influences Dothideomycete-Induced Diseae

**Figure 3:**
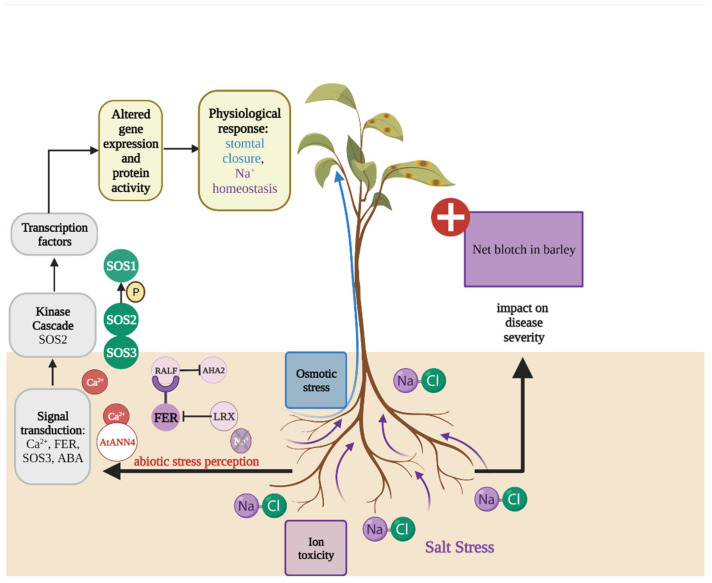
Overview of the salt stress signalling pathway in plants and effects of host salt stress on Dothideomycete-induced disease severity. Figure 3 highlights two pathways that are involved in the salt response leading to sodium (Na^+^) homeostasis (**left**) and the effect of salt stress on Dothideomycete-induced disease severity (**right**). In *A. thaliana*, the malectin receptor-like kinase FERONIA (FER)-pathway involves specific binding of rapid alkalisation factors (RALFs), which leads to FER phosphorylation and inhibition of plasma membrane H^+^-ATPase 2 (AHA2) and results in cell wall alkalisation. Salt stress-associated cell wall damage is sensed by the FER signalling pathway (purple) and is proposed to trigger transient cytosolic calcium ion (Ca^2+^) accumulation. In the absence of salt stress, leucine-rich-repeat extensins (LRXs) interact with RALFs and prevent the interaction with FER. The salt overly sensitive (SOS) pathway (green), which comprises a Ca^2+^-binding protein SOS3, leads to the export of Na^+^ from root epidermal cells to the soil via the Na^+^/H^+^ antiporter SOS1. SOS3 activates SOS2, a protein in the sucrose nonfermenting 1 (SNF1) related protein kinase 3 (SnRK3) family. At the plasma membrane, the SOS3-SOS2 complex phosphorylates SOS1 resulting in increased Na^+^ efflux. The Ca^2+^ permeable transporter AtANN4 in *A. thaliana* may form a negative feedback loop to fine tune the influx of Ca^2+^ in response to salt stress.

Globally over 800 million hectares of soil are affected by salt accumulation, which can have detrimental consequences for crop productivity [125]. According to the FAO, the loss of arable land attributed to increased salinisation will amount to up to 30% by 2050. Although some plant species known as halophytes have evolved mechanisms to thrive under salinity stress, most crop plants are glycophytes, i.e., salt-sensitive. Soil salinity can arise from climatic events such as wind, rain, or the weathering of rocks, but also from human activities [126]. Soil salinity causes water deficit due to low water potential, leading to osmotic pressure at the external root zone, toxicity caused by salt ions such as sodium (Na^+^) and chloride (Cl^−^), and reduced uptake of essential mineral nutrients due to excessive sodium and chloride ions in the soil [126,127,128]. This in turn negatively affects plant physiology due to ionic imbalances in cells, reduced nutrient uptake and production of excessive ROS [129]. The stress response to the accumulation of toxic ions such as Na^+^ has been described in *A. thaliana* and contributed to the characterisation of the salt overly sensitive (SOS) pathway that leads to the export of Na^+^ from root epidermal cells into the soil environment (Figure 3) [27,130,131]. To prevent cell damage due to osmotic stress, cell wall alkalisation is initiated. This process involves the malectin receptor-like kinase FERONIA (FER) pathway that binds rapid alkalisation factors (RALFs) leading to the inhibition of plasma proton (H^+^)-ATPase 2 (AHA2), preventing cells from bursting [132,133] (Figure 3). 

With regards to Dothideomycete-induced diseases, salt stress can exacerbate net blotch disease severity in barley. Net blotch is caused by the necrotrophic fungus *Drechslera teres* (teleomorph *Pyrenophora teres*) and is one of the most important diseases in barley growing areas of the world [134]. This disease causes yield losses between 10% and 40%, with the potential for total yield loss under extreme environmental conditions [135]. Moreover, an infection with *D. teres* can lead to a reduction in kernel weight, plumpness and bulk density, therefore negatively affecting grain quality [136]. Ben Alaya et al. [137] showed that the leaves of barley plants grown in a high salt concentration exhibited larger net blotch lesions compared to those of plants grown in a low salt concentration. Furthermore, an in vitro assay showed that the growth of several *D. teres* isolates was reduced in media supplemented with sodium chloride compared to salt-free media [137]. Taken together, these results suggest that the observed effect of salt on disease severity cannot be attributed to a direct effect of salt on fungal fitness, but rather, may be the result of salt-induced host stress. The authors hypothesised that the accumulation of ABA in response to salt stress might suppress signalling pathways involved in the response to biotic stresses, therefore contributing to the severity of net blotch in barley leaves subjected to high salt concentrations [137,138,139]. ABA is a phytohormone involved in the abiotic stress response, and high concentrations of ABA can repress SA- and JA-regulated gene expression, which can lead to a reduced resistance to biotic stressors [140,141]. These observations are in agreement with anecdotal reports from northern Egypt where net blotch appears to have become more severe in salinity-affected soils [142]. 

## 6. Light Stress Affects Dothideomycete-Induced Disease Development

**Figure 4:**
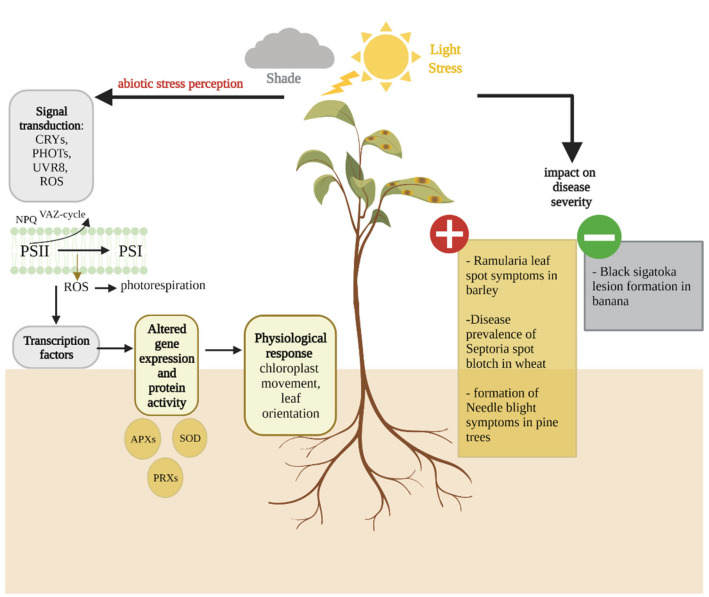
Overview of the host light stress response and its impact on Dothideomycete-induced disease severity. Figure 4 highlights the signalling pathway involved in the light stress response in the host (**left**) and the effect of high light (orange) and shade (grey) stresses on Dothideomycete-induced disease severity (**right**). Light intensity is perceived by UV-A/B photoreceptors, such as cryptochromes (CRYs), phototropins (PHOTs) and the UV RESISTANCE LOCUS 8 (UVR8). Excess light leads to photoinhibition of the photosystem II (PSII), resulting in the production of reactive oxygen species (ROS) in the chloroplasts. To prevent photoinhibition-associated damage, photoprotection mechanisms include cyclic electron flow (CEF), the xanthophyll cycle (violaxanthin-antheraxanthin-zeaxanthin cycle, VAZ), the photorespiratory pathway and ROS scavenging enzymes, such as ascorbate peroxidases (APXs), peroxiredoxins (PRXs) and superoxide dismutase (SOD). In response to high light, chloroplasts move away from the cell surface to reduce light absorption in a process known as chloroplast avoidance. In contrast, under low light conditions, chloroplasts accumulate near the cell surface.

With the exception of some parasitic plants, light is integral to the life of most plants. Light signals are required for breaking seed dormancy and seedling development. Light also provides energy for photosynthesis, as well as information about the circadian rhythm and the seasonal changes that regulate several plant physiological processes, such as leaf senescence [143,144]. However, light is also one of the most important sources of stress for plants worldwide, as variations in light intensity can lead to disruption of redox homeostasis in the chloroplast, which results in the accumulation of ROS that can damage cell membranes and proteins [145]. Light can induce stress in plants in several ways. Light fluctuation, i.e., sudden changes in the amount of light received by the plant, light intensity and UV light all have the potential to damage the photosystems and can therefore act as stressors [146]. As light plays a prominent role for plants, they have evolved several mechanisms to alleviate the effect of light stresses. These mechanisms have been recently reviewed in great detail elsewhere [53,147] and will not be discussed here. However, in a changing environment, these mechanisms are showing their limits. A recent study showed that with climate change, plants were frequently subjected to multifactorial stresses, i.e., a combination of several abiotic stresses, and that multifactorial stresses not only negatively affected plant growth and survival, but also damaged the microbiome supported by plants [148]. Considering that both the microbiome and the light signalling pathway are involved in response to biotic stresses, a strong link exists between light stress and disease development [149,150,151]. For instance, wheat seedlings stressed by high light intensity exhibit an increased susceptibility to the yellow rust pathogen *Puccinia striiformis* [152]. Light stress in the host is known to influence the development of diseases caused by several Dothideomycete fungi (Figure 4). 

In the barley pathogen *R. collo-cygni*, light stress in the host is known to influence disease development. Plants grown under controlled conditions and exposed to high light intensity prior to inoculation exhibited increased disease symptoms compared to plants exposed to low light intensity before inoculation [108,153]. Light stress in plants is linked with the production of ROS, including hydrogen peroxide [154]. McGrann and Brown [108] showed that barley seedlings infiltrated with hydrogen peroxide during the asymptomatic growth of *R. collo-cygni* exhibited increased disease levels, suggesting that ROS may play an important role in the transition from asymptomatic to symptomatic growth. Interestingly, the development of RLS symptoms has historically been linked with the release and action of the non-host-specific, light-activated toxin rubellin D by *R. collo-cygni* in its host, since plant cell death was observed away from fungal hyphae. Heiser et al. [155] showed that rubellin D, one of the rubellin derivatives, is able to induce ROS production in vitro. Furthermore, Dussart et al. [156] showed that most of the polyketide synthase (PKS) genes predicted to play a key role in secondary metabolite biosynthesis, including rubellin D in *R. collo-cygni*, were highly expressed during asymptomatic growth of the fungus in the host. Recently, Dussart and Jakubczyk [157] showed that all of the genes in the rubellin candidate biosynthetic gene cluster are predominantly expressed during the asymptomatic and early symptom development stages of RLS development in artificially inoculated barley seedlings. Taken together, these results suggest that light may play an important role in RLS development, be it directly through abiotic stress-induced ROS production, or through its role in rubellin D activation.

Environmental conditions are known to affect the development of the wheat pathogen *M. graminicola*. Daamen and Stol [158] showed a positive correlation between hours of sunshine and disease prevalence in the field; however, the cause for that relationship cannot be solely attributed to host stress. The authors hypothesised that increased temperature linked with long sunshine hours could result in slower growth of saprophytic fungi in the straw, leaving more nutrients available for *M. graminicola*. Detached leaf assays carried out by Arraiano et al. [159] support the role of light in disease development, as a positive correlation was found between symptom levels and light levels, since near UV lights resulted in increased disease levels. However, the experiment simultaneously investigated the effect of temperature, and it is therefore difficult to attribute these effects to light stress in the host.

Light also plays a role in the development of other Dothideomycete-induced diseases, including black Sigatoka and red band needle blight in banana plants and various species of pine trees, respectively. Banana plants grown under shade exhibit smaller lesions than plants grown under full sunlight [22]. Similarly, high light intensity increases the formation of red band needle blight symptoms [160]. However, it is difficult to ascertain the role played by light stress in the host during the development of these diseases as both *Pseudocercospora fijiensis* and *Dothistroma septosporum*, the pathogens responsible for black Sigatoka and red band needle blight, respectively, produce photoactivated toxic metabolites. Dothistromin is a light-activated anthraquinone synthesised by *D. septosporum* that acts as a virulence factor during disease development [161,162]. Mutants of *D. septosporum* with impaired dothistromin production induced smaller lesions than the wild type [162]. This suggests that the effect of light on red band needle blight development might be due to the increased activity of the dothistromin toxin rather than light stress in the host. Similarly, juglone is a light-activated metabolite produced by *P. fijiensis* that has been hypothesised to be responsible for the effect of light on the development of black Sigatoka, as susceptibility to juglone appears to be correlated with disease susceptibility [163]. However, the effect of shade on black Sigatoka was also observed for yellow Sigatoka caused by *Pseudocercospora musae* despite the absence of juglone production by the pathogen. Taken together, these results suggest that the light effect cannot be explained solely by the action of juglone in the plant. The role played by juglone in the interactions between *P. fijiensis* and banana has been reviewed in this issue by Noar et al. [164].

It is important to note that many other Dothideomycete fungi that cause plant diseases are able to produce light-activated toxins. Cercosporin and altertoxins are light-activated perylenequinone secondary metabolites produced by several fungi in the *Cercospora* and *Alternaria* genus, respectively [165,166]. Similarly, *Cladosporium phlei*, the pathogen responsible for purple spot disease in timothy grass (*Phleum pratense*), produces phleichrome, another light-activated perylenequinone secondary metabolite [167]. Cercosporin is arguably the most studied non-host-specific phytotoxin and is known to play an important role in the development of Cercospora leaf spot diseases in various plant species [166,168,169]. The mode of action of this toxin was first studied in the mid 1970s [170] and involves the production of ROS singlet oxygen and superoxide as a result of light activation, leading to the classification of cercosporin as a photosensitiser toxin [171]. Perylenequinones are known to act as photosensitisers, being able to transform light energy into chemical energy through the production of ROS. Given that ROS production is also associated with the response to light stress in plants, distinguishing between the role of host stress and the action of the toxin in disease development is difficult in many cases. 

## 7. Conclusions

This review mainly focused on how abiotic stresses in the host affect its interaction with plant pathogenic Dothideomycetes. It appears clearly that in most cases, plants subjected to an abiotic stress, or to a combination of abiotic stresses, are more susceptible to diseases than plants grown in the absence of abiotic stress (Table 1). However, it is important to note that abiotic stress factors, such as changing rainfall patterns or increased temperature, can also contribute to a change in the fitness and aggressiveness of fungal pathogens. The lack of studies investigating the mechanisms underlying altered disease susceptibility as a response to abiotic stresses makes it difficult to disentangle the effect of the stress on the host and the effect of environmental conditions on the pathogen. Nevertheless, the observation that host abiotic stress favours the development of many Dothideomycetes-induced diseases is particularly worrying in the context of the current climate crisis. To date, crop migration has mitigated the adverse effect of climate change on yield in major crops such as maize, rice or wheat [172]. However, crop migration has substantial limitations, including environmental, social and economic costs, as it is often accompanied with reduced biodiversity, carbon release from the soil and increased irrigation. Furthermore, land availability and suitability are important limiting factors to crop migration. Therefore, to be most effective, this strategy will need to be integrated with other strategies to alleviate the stress incurred by climate change. Among the diverse strategies to limit the effect of climate change-associated abiotic stress on crop productivity, breeding for stress tolerance is seen as a step towards safeguarding food security. However, plants are often subjected to several stress factors and, in a changing climate with increased air pollution, it is likely that plants will face multifactorial abiotic stresses. In addition, as highlighted in this review, abiotic stresses may be accompanied by increased biotic pressure on crops. Therefore, understanding how plant-pathogen interactions are affected at the molecular level by abiotic stresses on the host may be a crucial step towards developing multifactorial stress-resistant or stress-tolerant crops to maintain crop productivity.

## Figures and Tables

**Table 1:** Effect of selected abiotic stresses on plant-pathogenic Dothideomycete interactions.

Stress	Pathogen	Disease/Crop	Effect on Disease Development	References
Heat	*Bipolaris sorokiniana* (*Cochliobolus sativus)*	Spot blotch in cereals	Increase in spot blotch	[57,58,59,62,63,64,65]
	*Ramularia collo-cygni*	Ramularia leaf spot (RLS) in barley	Decrease in RLS symptom development	[81,82]
	*Parastagnorospora nodorum* (*Phaeosphaeria nodorum*)	Septoria nodrum blotch and glume blotch in wheat	Reduced *P. nodrum* DNA in winter wheat grains	[71,72,75]
	Phoma lingam (*Leptosphaeria maculans*)	Blackleg and Phoma stem canker in oilseed rape	Increased lesion formation	[91]
Cold	*Alternaria spp.* *(A. alternata* and *A. macrospora)*	Leaf spot in cotton	Increase in leaf spot	[85]
	*Ascochyta rabiei* (*Didymella rabiei*)	Ascochyta blight in chickpea	Increase in Ascochyta blight symptoms	[86]
Waterlogging & Flooding	*Ramularia collo-cygni*	RLS in barley	Increase in RLS symptom development	[119,120]
Drought	*Ramularia collo-cygni*	RLS in barley	Drought led to increased resistance to RLS	[80,82]
	*Macrophomina phaseolina*	Dry root rot (DRR) in chickpea	Drought led to enhanced DRR infection and colonisation	[117]
Salinity	*Drechslera teres* (*Pyrenophora teres*)	Net blotch in barley	High salt concentration led to increase in net blotch	[137,142]
Light	*Ramularia collo-cygni*	RLS in barley	Increased RLS disease symptoms	[120,162]
	*Zymoseptoria tritici (Mycosphaerella graminicola)*	Septoria tritici blotch in wheat	Positive correlation between hours of sunshine and disease prevalence	[158,159]
	*Dothistroma septosporum* (*Mycosphaerella pini*)	Needle blight in various pine trees	Light intensity increased formation of symptoms	[160,161,162]
	*Pseudocercospora fijiensis* *(Mycosphaerella fijiensis*)	Black Sigatoka disease in banana	Shade decreases black Sigatoka lesion formation	[22]

## Data Availability

Not applicable.

## References

[1] Bebber D.P., Ramotowski M.A.T., Gurr S.J. (2013). Crop pests and pathogens move polewards in a warming world. Nat. Clim. Chang..

[2] Chaloner T.M., Gurr S.J., Bebber D.P. (2021). Plant pathogen infection risk tracks global crop yields under climate change. Nat. Clim. Chang..

[3] Pereira A. (2016). Plant abiotic stress challenges from the changing environment. Front. Plant Sci..

[4] Wang W., Vinocur B., Altman A. (2003). Plant responses to drought, salinity and extreme temperatures: Towards genetic engineering for stress tolerance. Planta.

[5] Godoy F., Olivos-Hernández K., Stange C., Handford M. (2021). Abiotic stress in crop species: Improving tolerance by applying plant metabolites. Plants.

[6] Munns R., Tester M. (2008). Mechanisms of salinity tolerance. Annu. Rev. Plant Biol..

[7] Atafar Z., Mesdaghinia A., Nouri J., Homaee M., Yunesian M., Ahmadimoghaddam M., Mahvi A.H. (2010). Effect of fertilizer application on soil heavy metal concentration. Environ. Monit. Assess..

[8] Manzoor J., Sharma M., Wani K.A. (2018). Heavy metals in vegetables and their impact on the nutrient quality of vegetables: A review. J. Plant Nutr..

[9] Snowdon R.J., Wittkop B., Chen T.W., Stahl A. (2021). Crop adaptation to climate change as a consequence of long-term breeding. Theor. Appl. Genet..

[10] Redman R.S., Kim Y.O., Woodward C.J.D.A., Greer C., Espino L., Doty S.L., Rodriguez R.J. (2011). Increased fitness of rice plants to abiotic stress via habitat adapted symbiosis: A strategy for mitigating impacts of climate change. PLoS ONE.

[11] Lohani N., Jain D., Singh M.B., Bhalla P.L. (2020). Engineering Multiple Abiotic Stress Tolerance in Canola, *Brassica napus*. Front. Plant Sci..

[12] Chojak-Koźniewska J., Kuźniak E., Zimny J. (2018). The effects of combined abiotic and pathogen stress in plants: Insights from salinity and *Pseudomonas syringae pv lachrymans* interaction in cucumber. Front. Plant Sci..

[13] Vaughan M.M., Huffaker A., Schmelz E.A., Dafoe N.J., Christensen S.A., McAuslane H.J., Alborn H.T., Allen L.H., Teal P.E.A. (2016). Interactive effects of elevated [CO_2_] and drought on the maize phytochemical defense response against mycotoxigenic *Fusarium verticillioides*. PLoS ONE.

[14] Sewelam N., El-Shetehy M., Mauch F., Maurino V.G. (2021). Combined abiotic stresses repress defense and cell wall metabolic genes and render plants more susceptible to pathogen infection. Plants.

[15] Creelman R.A., Mullet J.E. (1995). Jasmonic acid distribution and action in plants: Regulation during development and response to biotic and abiotic stress. Proc. Natl. Acad. Sci. USA.

[16] Horváth E., Szalai G., Janda T. (2007). Induction of abiotic stress tolerance by salicylic acid signaling. J. Plant Growth Regul..

[17] Huot B., Castroverde C.D.M., Velásquez A.C., Hubbard E., Pulman J.A., Yao J., Childs K.L., Tsuda K., Montgomery B.L., He S.Y. (2017). Dual impact of elevated temperature on plant defence and bacterial virulence in *Arabidopsis*. Nat. Commun..

[18] Vaughan M.M., Huffaker A., Schmelz E.A., Dafoe N.J., Christensen S., Sims J., Martins V.F., Swerbilow J., Romero M., Alborn H.T. (2014). Effects of elevated [CO_2_] on maize defence against mycotoxigenic *Fusarium verticillioides*. Plant Cell Environ..

[19] Wijayawardene N.N., Hyde K.D., Rajeshkumar K.C., Hawksworth D.L., Madrid H., Kirk P.M., Braun U., Singh R.V., Crous P.W., Kukwa M. (2017). Notes for genera: Ascomycota. Fungal Divers..

[20] Tiley A.M., Karki S.J., Feechan A. (2018). Zymoseptoria Tritici. eLS.

[21] See P.T., Schultz N., Moffat C.S. (2020). Evaluation of *Pyrenophora tritici-repentis* infection of wheat heads. Agriculture.

[22] Churchill A.C.L. (2011). Mycosphaerella fijiensis, the black leaf streak pathogen of banana: Progress towards understanding pathogen biology and detection, disease development, and the challenges of control. Mol. Plant Pathol..

[23] Noar R.D., Thomas E., Daub M.E. (2019). A novel polyketide synthase gene cluster in the plant pathogenic fungus *Pseudocercospora fijiensis*. PLoS ONE.

[24] Westman S.M., Kloth K.J., Hanson J., Ohlsson A.B., Albrectsen B.R. (2019). Defence priming in *Arabidopsis*—A Meta-Analysis. Sci. Rep..

[25] Atkinson N.J., Urwin P.E. (2012). The interaction of plant biotic and abiotic stresses: From genes to the field. J. Exp. Bot..

[26] Zhang H., Sonnewald U. (2017). Differences and commonalities of plant responses to single and combined stresses. Plant J..

[27] Gong Z., Xiong L., Shi H., Yang S., Herrera-Estrella L.R., Xu G., Chao D.Y., Li J., Wang P.Y., Qin F. (2020). Plant abiotic stress response and nutrient use efficiency. Sci. China Life Sci..

[28] Luan S. (2009). The CBL-CIPK network in plant calcium signaling. Trends Plant Sci..

[29] Yang T., Poovaiah B.W. (2003). Calcium/calmodulin-mediated signal network in plants. Trends Plant Sci..

[30] Fichman Y., Mittler R. (2020). Rapid systemic signaling during abiotic and biotic stresses: Is the ROS wave master of all trades?. Plant J..

[31] Cheng S.H., Willmann M.R., Chen H.C., Sheen J. (2002). Calcium signaling through protein kinases. The *Arabidopsis* calcium-dependent protein kinase gene family. Plant Physiol..

[32] Liang X., Zhou J.M. (2018). Receptor-Like Cytoplasmic Kinases: Central Players in Plant Receptor Kinase-Mediated Signaling. Annu. Rev. Plant Biol..

[33] Nephali L., Piater L.A., Dubery I.A., Patterson V., Huyser J., Burgess K., Tugizimana F. (2020). Biostimulants for Plant Growth and Mitigation of Abiotic Stresses: A Metabolomics Perspective. Metabolites.

[34] Sandrini M., Nerva L., Sillo F., Balestrini R., Chitarra W., Zampieri E. (2022). Abiotic Stress and Belowground Microbiome: The Potential of Omics Approaches. Int. J. Mol. Sci..

[35] Qi J., Song C.P., Wang B., Zhou J., Kangasjärvi J., Zhu J.K., Gong Z. (2018). Reactive oxygen species signaling and stomatal movement in plant responses to drought stress and pathogen attack. J. Integr. Plant Biol..

[36] Khan N., Bano A., Ali S., Babar M.A. (2020). Crosstalk amongst phytohormones from planta and PGPR under biotic and abiotic stresses. Plant Growth Regul..

[37] Santner A., Calderon-Villalobos L.I.A., Estelle M. (2009). Plant hormones are versatile chemical regulators of plant growth. Nat. Chem. Biol..

[38] Choudhury F.K., Rivero R.M., Blumwald E., Mittler R. (2017). Reactive oxygen species, abiotic stress and stress combination. Plant J..

[39] Arbona V., Manzi M., Zandalinas S.I., Vives-Peris V., Pérez-Clemente R.M., Gómez-Cadenas A. (2016). Physiological, metabolic, and molecular responses of plants to abiotic stress. Stress Signaling in Plants: Genomics and Proteomics Perspective.

[40] Pieterse C.M.J., Van der Does D., Zamioudis C., Leon-Reyes A., Van Wees S.C.M. (2012). Hormonal Modulation of Plant Immunity. Annu. Rev. Cell Dev. Biol..

[41] Miller G.A.D., Suzuki N., Ciftci-Yilmaz S.U.L.T.A.N., Mittler R.O.N. (2010). Reactive oxygen species homeostasis and signalling during drought and salinity stresses. Plant. Cell Environ..

[42] Halliwell B., Gutteridge J. (2015). Free Radicals in Biology and Medicine.

[43] Suzuki N., Koussevitzky S., Mittler R., Miller G. (2012). ROS and redox signalling in the response of plants to abiotic stress. Plant Cell Environ..

[44] Mittler R., Vanderauwera S., Gollery M., Van Breusegem F. (2004). Reactive oxygen gene network of plants. Trends Plant Sci..

[45] Rizhsky L., Liang H., Shuman J., Shulaev V., Davletova S., Mittler R. (2004). When defense pathways collide. The response of arabidopsis to a combination of drought and heat stress. Plant Physiol..

[46] Mittler R., Blumwald E. (2010). Genetic engineering for modern agriculture: Challenges and perspectives. Annu. Rev. Plant Biol..

[47] Nagarajan S., Nagarajan S. (2010). Abiotic tolerance and crop improvement. Abiotic Stress Adaptation in Plants: Physiological, Molecular and Genomic Foundation.

[48] Ranty B., Aldon D., Cotelle V., Galaud J.P., Thuleau P., Mazars C. (2016). Calcium sensors as key hubs in plant responses to biotic and abiotic stresses. Front. Plant Sci..

[49] Gao F., Han X., Wu J., Zheng S., Shang Z., Sun D., Zhou R., Li B. (2012). A heat-activated calcium-permeable channel–Arabidopsis cyclic nucleotide-gated ion channel 6–is involved in heat shock responses. Wiley Online Libr..

[50] Ohama N., Sato H., Shinozaki K., Yamaguchi-Shinozaki K. (2017). Transcriptional Regulatory Network of Plant Heat Stress Response. Trends Plant Sci..

[51] Jaglo-Ottosen K.R., Gilmour S.J., Zarka D.G., Schabenberger O., Thomashow M.F. (1998). Arabidopsis *CBF1* Overexpression Induces COR Genes and Enhances Freezing Tolerance. Science.

[52] Hasanuzzaman M., Nahar K., Alam M.M., Roychowdhury R., Fujita M. (2013). Physiological, biochemical, and molecular mechanisms of heat stress tolerance in plants. Int. J. Mol. Sci..

[53] Szymańska R., Ślesak I., Orzechowska A., Kruk J. (2017). Physiological and biochemical responses to high light and temperature stress in plants. Environ. Exp. Bot..

[54] Harding S.A., Guikema J.A., Paulsen G.M. (1990). Photosynthetic decline from high temperature stress during maturation of wheat: I. Interaction with senescence processes. Plant Physiol..

[55] Bheemanahalli R., Sunoj V.S.J., Saripalli G., Prasad P.V.V., Balyan H.S., Gupta P.K., Grant N., Gill K.S., Jagadish S.V.K. (2019). Quantifying the impact of heat stress on pollen germination, seed set, and grain filling in spring wheat. Crop Sci..

[56] Aiqing S., Somayanda I., Sebastian S.V., Singh K., Gill K., Prasad P.V.V., Jagadish S.V.K. (2018). Heat Stress during Flowering Affects Time of Day of Flowering, Seed Set, and Grain Quality in Spring Wheat. Crop Sci..

[57] Cheng C., Gao X., Feng B., Sheen J., Shan L., He P. (2013). Plant immune response to pathogens differs with changing temperatures. Nat. Commun..

[58] Gupta P.K., Chand R., Vasistha N.K., Pandey S.P., Kumar U., Mishra V.K., Joshi A.K. (2018). Spot blotch disease of wheat: The current status of research on genetics and breeding. Wiley Online Libr..

[59] Dubin H.J., Van Ginkel M. (1991). The status of wheat diseases and disease research in warmer areas. Wheat for the Nontraditional Warm Areas: In Proceedings of the International Conference, Foz do Iguacu, Brazil, 29 July–3 August 1990.

[60] Duveiller E., Dubin H.J., Reeves J., McNab A. (1997). Helminthosporium Blights of Wheat: Spot Blotch and Tan Spot.

[61] Joshi A.K., Mishra B., Chatrath R., Ortiz Ferrara G., Singh R.P. (2007). Wheat improvement in India: Present status, emerging challenges and future prospects. Euphytica.

[62] Joshi A.K., Ortiz-Ferrara G., Crossa J., Singh G., Sharma R.C., Chand R., Parsad R. (2007). Combining superior agronomic performance and terminal heat tolerance with resistance to spot blotch (*Bipolaris sorokiniana*) of wheat in the warm humid Gangetic Plains of South Asia. Field Crops Res..

[63] Chaurasia S., Chand R., Joshi A.K. (2000). Relative dominance of *Alternaria triticina* Pras. et Prab. and *Bipolaris sorokiniana* (Sacc.) Shoemaker in different growth stages of wheat (*T. aestivum L.*). Z. Fur Pflanzenkrankh. Und Pflanzenschutz.

[64] Sharma R.C., Duveiller E., Ortiz-Ferrara G. (2007). Progress and challenge towards reducing wheat spot blotch threat in the Eastern Gangetic Plains of South Asia: Is climate change already taking its toll?. Field Crops Res..

[65] Mercado Vergnes D., Renard M.E., Duveiller E., Maraite H. (2006). Effect of growth stage on host sensitivity to helminthosporol toxin and susceptibility to *Cochliobolus sativus* causing spot blotch on wheat. Physiol. Mol. Plant Pathol..

[66] Schützendübel A., Stadler M., Wallner D., Von Tiedemann A. (2008). A hypothesis on physiological alterations during plant ontogenesis governing susceptibility of winter barley to ramularia leaf spot. Plant Pathol..

[67] Havis N.D., Brown J.K.M., Clemente G., Frei P., Jedryczka M., Kaczmarek J., Kaczmarek M., Matusinsky P., McGrann G.R.D., Piotrowska M. (2015). *Ramularia collo-cygni*—An emerging pathogen of barley crops. Phytopathology.

[68] McGrann G.R.D., Steed A., Burt C., Goddard R., Lachaux C., Bansal A., Corbitt M., Gorniak K., Nicholson P., Brown J.K.M. (2015). Contribution of the drought tolerance-related Stress-responsive NAC1 transcription factor to resistance of barley to Ramularia leaf spot. Mol. Plant Pathol..

[69] Mařík P., Šnejdar Z., Matušinsky P. (2011). Expression of resistance to ramularia leaf spot in winter barley cultivars grown in conditions of the Czech Republic. Czech J. Genet. Plant Breed..

[70] Hoheneder F., Hofer K., Groth J., Herz M., Heß M., Hückelhoven R. (2021). Ramularia leaf spot disease of barley is highly host genotype-dependent and suppressed by continuous drought stress in the field. J. Plant Dis. Prot..

[71] Syme R.A., Tan K.C., Hane J.K., Dodhia K., Stoll T., Hastie M., Furuki E., Ellwood S.R., Williams A.H., Tan Y.F. (2016). Comprehensive Annotation of the *Parastagonospora nodorum* Reference Genome Using Next-Generation Genomics, Transcriptomics and Proteogenomics. PLoS ONE.

[72] Shaw M.W., Bearchell S.J., Fitt B.D.L., Fraaije B.A. (2008). Long-term relationships between environment and abundance in wheat of *Phaeosphaeria nodorum* and *Mycosphaerella graminicola*. New Phytol..

[73] Downie R.C., Lin M., Corsi B., Ficke A., Lillemo M., Oliver R.P., Phan H.T.T., Tan K.C., Cockram J. (2021). Septoria nodorum blotch of wheat: Disease management and resistance breeding in the face of shifting disease dynamics and a changing environment. Phytopathology.

[74] Bhathal J.S., Loughman R., Speijers J. (2003). Yield reduction in wheat in relation to leaf disease from yellow (tan) spot and septoria nodorum blotch. Eur. J. Plant Pathol..

[75] Bishaw Z., Struik P.C., Van Gastel A.J.G. (2013). Farmer’s seed sources and seed quality: 2. seed health. Int. J. Plant Prod..

[76] Yang C., Zou Z., Fernando W.G.D. (2021). The Effect of Temperature on the Hypersensitive Response (HR) in the *Brassica napus*–*Leptosphaeria maculans* Pathosystem. Plants.

[77] Huang Y.J., Evans N., Li Z.Q., Eckert M., Chèvre A.M., Renard M., Fitt B.D. (2006). Temperature and leaf wetness duration affect phenotypic expression of Rlm6-mediated resistance to Leptosphaeria maculans in Brassica napus. New Phytol..

[78] West J.S., Kharbanda P.D., Barbetti M.J., Fitt B.D.L. (2001). Epidemiology and management of *Leptosphaeria maculans* (phoma stem canker) on oilseed rape in Australia, Canada and Europe. Plant Pathol..

[79] Thuiller W., Lavorel S., Araújo M.B., Sykes M.T., Prentice I.C. (2005). Climate change threats to plant diversity in Europe. Proc. Natl. Acad. Sci. USA.

[80] Nurhasanah Ritonga F., Chen S. (2020). Physiological and molecular mechanism involved in cold stress tolerance in plants. Plants.

[81] Levitt J. (1980). Responses of Plants to Environmental Stresses. Volume II. Water, Radiation, Salt, and Other Stresses.

[82] John R., Anjum N.A., Sopory S.K., Akram N.A., Ashraf M. (2016). Some key physiological and molecular processes of cold acclimation. Biol. Plant..

[83] Chen L.J., Xiang H.Z., Miao Y., Zhang L., Guo Z.F., Zhao X.H., Lin J.W., Li T.L. (2014). An Overview of Cold Resistance in Plants. J. Agron. Crop Sci..

[84] Lucau-Danila A., Toitot C., Goulas E., Blervacq A.S., Hot D., Bahrman N., Sellier H., Lejeune-Hénaut I., Delbreil B. (2012). Transcriptome analysis in pea allows to distinguish chilling and acclimation mechanisms. Plant Physiol. Biochem..

[85] Zhao J., Li S., Jiang T., Liu Z., Zhang W., Jian G., Qi F. (2012). Chilling stress-the key predisposing factor for causing *alternaria alternata* infection and leading to cotton (*Gossypium hirsutum* L.) leaf senescence. PLoS ONE.

[86] Kemal S.A., Bencheqroun S.K., Hamwieh A., Imtiaz M. (2017). Effects of temperature stresses on the resistance of chickpea genotypes and aggressiveness of *Didymella rabiei* isolates. Front. Plant Sci..

[87] Bashan Y., Hernandez-Saavedra N.Y., Chelkowski J., Visconti A. (1992). Alternaria-blight of cotton: Epidemiology and transmission. Alternaria Biology, Plant Diseases and Metabolites.

[88] Bashi E., Sachs Y., Rotem J. (1983). Relationships between disease and yield in cotton fields affected by *Alternaria macrospora*. Phytoparasitica.

[89] Muehlbauer F.J., Chen W. (2007). Resistance to ascochyta blights of cool season food legumes. Ascochyta Blights Grain Legum..

[90] Singh K., Nizam S., Sinha M., Verma P.K. (2012). Comparative Transcriptome Analysis of the Necrotrophic Fungus *Ascochyta rabiei* during Oxidative Stress: Insight for Fungal Survival in the Host Plant. PLoS ONE.

[91] Nene Y.L. (1982). A review of Ascochyta blight of chickpea. Trop. Pest Manag..

[92] Christensen J.H., Hewitson B., Busuioc A., Chen A., Gao X., Held I., Jones R., Kolli R.K., Kwon W.-T., Laprise R. (2007). Regional Climate Projections—Supplementary Material.

[93] Fukao T., Bailey-Serres J. (2008). Submergence tolerance conferred by *Sub1A* is mediated by SLR1 and SLRL1 restriction of gibberellin responses in rice. Proc. Natl. Acad. Sci. USA.

[94] Hoffmann-Benning S., Kende H. (1992). On the Role of Abscisic Acid and Gibberellin in the Regulation of Growth in Rice. Plant Physiol..

[95] Ma Y., Szostkiewicz I., Korte A., Moes D., Yang Y., Christmann A., Grill E. (2009). Regulators of PP2C phosphatase activity function as abscisic acid sensors. Science.

[96] Melcher K., Ng L.M., Zhou X.E., Soon F.F., Xu Y., Suino-Powell K.M., Park S.Y., Weiner J.J., Fujii H., Chinnusamy V. (2009). A gate–latch–lock mechanism for hormone signalling by abscisic acid receptors. Nature.

[97] Palmer T.N., Räisänen J. (2002). Quantifying the risk of extreme seasonal precipitation events in a changing climate. Nature.

[98] Christensen J.H., Christensen O.B. (2003). Severe summertime flooding in Europe. Nature.

[99] Hemantaranjan A. (2014). Flooding: Abiotic Constraint Limiting Vegetable Productivity. Adv. Plants Agric. Res..

[100] Tewari S., Mishra A., Ahmad P., Ahanger M.A., Singh V.P., Tripathi D.K., Alam P., Alyemeni M.N. (2018). Flooding Stress in Plants and Approaches to Overcome. Plant Metab. Regul. Under Environ. Stress.

[101] Parent C., Capelli N., Berger A., Crèvecoeur M., Dat J.F. (2008). An overview of plant responses to soil waterlogging. Plant Stress.

[102] Lu Y., Watanabe A., Kimura M. (2004). Contribution of plant photosynthates to dissolved organic carbon in a flooded rice soil. Biogeochemistry.

[103] Kreuzwieser J., Rennenberg H. (2014). Molecular and physiological responses of trees to waterlogging stress. Plant Cell Environ..

[104] Irfan M., Hayat S., Hayat Q., Afroz S., Ahmad A. (2010). Physiological and biochemical changes in plants under waterlogging. Protoplasma.

[105] Verma V., Ravindran P., Kumar P.P. (2016). Plant hormone-mediated regulation of stress responses. BMC Plant Biol..

[106] Havis N.D., Nyman M., Oxley S.J.P. (2014). Evidence for seed transmission and symptomless growth of *Ramularia collo-cygni* in barley (*Hordeum vulgare*). Plant Pathol..

[107] McGrann G.R.D., Havis N.D. (2017). Ramularia leaf spot: A newly important threat to barley production. Outlooks Pest Manag..

[108] McGrann G.R.D., Brown J.K.M. (2018). The role of reactive oxygen in the development of Ramularia leaf spot disease in barley seedlings. Ann. Bot..

[109] Thornton P.K., Ericksen P.J., Herrero M., Challinor A.J. (2014). Climate variability and vulnerability to climate change: A review. Glob. Chang. Biol..

[110] Alamri S., Hu Y., Mukherjee S., Aftab T., Fahad S., Raza A., Ahmad M., Siddiqui M.H. (2020). Silicon-induced postponement of leaf senescence is accompanied by modulation of antioxidative defense and ion homeostasis in mustard (*Brassica juncea*) seedlings exposed to salinity and drought stress. Plant Physiol. Biochem..

[111] Seleiman M.F., Al-Suhaibani N., Ali N., Akmal M., Alotaibi M., Refay Y., Dindaroglu T., Abdul-Wajid H.H., Battaglia M.L. (2021). Drought stress impacts on plants and different approaches to alleviate its adverse effects. Plants.

[112] Guan X.K., Song L., Wang T.C., Turner N.C., Li F.M. (2015). Effect of Drought on the Gas Exchange, Chlorophyll Fluorescence and Yield of Six Different-Era Spring Wheat Cultivars. J. Agron. Crop Sci..

[113] Loggini B., Scartazza A., Brugnoli E., Navari-Izzo F. (1999). Antioxidative Defense System, Pigment Composition, and Photosynthetic Efficiency in Two Wheat Cultivars Subjected to Drought. Plant Physiol..

[114] Allu A.D., Soja A.M., Wu A., Szymanski J., Balazadeh S. (2014). Salt stress and senescence: Identification of cross-talk regulatory components. J. Exp. Bot..

[115] Hu H., Dai M., Yao J., Xiao B., Li X., Zhang Q., Xiong L. (2006). Overexpressing a NAM, ATAF, and CUC (NAC) transcription factor enhances drought resistance and salt tolerance in rice. Proc. Natl. Acad. Sci. USA.

[116] You J., Zong W., Li X., Ning J., Hu H., Li X., Xiao J., Xiong L. (2013). The SNAC1-targeted gene *OsSRO1c* modulates stomatal closure and oxidative stress tolerance by regulating hydrogen peroxide in rice. J. Exp. Bot..

[117] Irulappan V., Kandpal M., Saini K., Rai A., Ranjan A., Sinharoy S., Senthil-Kumar M. (2022). Drought stress exacerbates fungal colonization and endodermal invasion and dampens defense responses to increase dry root rot in chickpea. Mol. Plant Microbe Interact..

[118] Marquez N., Giachero M.L., Declerck S., Ducasse D.A. (2021). *Macrophomina phaseolina*: General Characteristics of Pathogenicity and Methods of Control. Front. Plant Sci..

[119] Schroeder M.M., Lai Y., Shirai M., Alsalek N., Tsuchiya T., Roberts P., Eulgem T. (2019). A novel *Arabidopsis* pathosystem reveals cooperation of multiple hormonal response-pathways in host resistance against the global crop destroyer *Macrophomina phaseolina*. Sci. Rep..

[120] Singh S.K. (1990). Influence of Cropping Systems on *Macrophomina phaseolina* Populations in Soil. Plant Dis..

[121] Kaur S., Dhillon G.S., Brar S.K., Vallad G.E., Chand R., Chauhan V.B. (2012). Emerging phytopathogen *Macrophomina phaseolina*: Biology, economic importance and current diagnostic trends. Crit. Rev. Microbiol..

[122] Mengistu A., Ray J.D., Smith J.R., Arelli P.R., Bellaloui N., Chen P., Shannon G., Boykin D. (2018). Effect of charcoal rot on selected putative drought tolerant soybean genotypes and yield. Crop Prot..

[123] Mihail J.D. (1989). *Macrophomina phaseolina*: Spatio-temporal dynamics of inoculum and of disease in a highly susceptible crop. Phytopathology.

[124] Goudarzi A., Banihashemi Z., Maftoun M. (2008). Effect of water potential on sclerotial germination and mycelial growth of *Macrophomina phaseolina*. Phytopathol. Mediterr..

[125] Rengasamy P. (2010). Soil processes affecting crop production in salt-affected soils. Funct. Plant Biol..

[126] Paul K., Pauk J., Kondic-Spika A., Grausgruber H., Allahverdiyev T., Sass L., Vass I. (2019). Co-occurrence of mild salinity and drought synergistically enhances biomass and grain retardation in wheat. Front. Plant Sci..

[127] Nedjimi B. (2015). Salinity tolerance: Growth, mineral nutrients, and roles of organic osmolytes, case of *Lygeum spartum* L., A review. Osmolytes and Plants Acclimation to Changing Environment: Emerging Omics Technologies.

[128] Arzani A., Ashraf M. (2016). Smart Engineering of Genetic Resources for Enhanced Salinity Tolerance in Crop Plants. CRC Crit. Rev. Plant Sci..

[129] Mushtaq Z., Faizan S., Gulzar B. (2020). Salt stress, its impacts on plants and the strategies plants are employing against it: A review. J. Appl. Biol. Biotechnol..

[130] Zhu J.K. (2001). Cell signaling under salt, water and cold stresses. Curr. Opin. Plant Biol..

[131] Liu J., Ishitani M., Halfter U., Kim C.S., Zhu J.K. (2000). The Arabidopsis thaliana *SOS2* gene encodes a protein kinase that is required for salt tolerance. Proc. Natl. Acad. Sci. USA.

[132] Haruta M., Sabat G., Stecker K., Minkoff B.B., Sussman M.R. (2014). A peptide hormone and its receptor protein kinase regulate plant cell expansion. Science.

[133] Feng W., Kita D., Peaucelle A., Cartwright H.N., Doan V., Duan Q., Liu M.C., Maman J., Steinhorst L., Schmitz-Thom I. (2018). The FERONIA Receptor Kinase Maintains Cell-Wall Integrity during Salt Stress through Ca^2+^ Signaling. Curr. Biol..

[134] Liu Z., Ellwood S.R., Oliver R.P., Friesen T.L. (2011). *Pyrenophora teres*: Profile of an increasingly damaging barley pathogen. Mol. Plant Pathol..

[135] Murray G.M., Brennan J.P. (2010). Estimating disease losses to the Australian barley industry. Australas. Plant Pathol..

[136] Grewal T.S., Rossnagel B.G., Pozniak C.J., Scoles G.J. (2008). Mapping quantitative trait loci associated with barley net blotch resistance. Theor. Appl. Genet..

[137] Ben Alaya A., Rabhi F., Hessini K., Djébali N. (2021). *Pyrenophora teres* growth and severity of net blotch on barley under salt stress. Eur. J. Plant Pathol..

[138] Mauch-Mani B., Mauch F. (2005). The role of abscisic acid in plant-pathogen interactions. Curr. Opin. Plant Biol..

[139] Ben Rejeb I., Pastor V., Mauch-Mani B. (2014). Plant Responses to Simultaneous Biotic and Abiotic Stress: Molecular Mechanisms. Plants.

[140] Gorshkov V., Tsers I. (2022). Plant susceptible responses: The underestimated side of plant–pathogen interactions. Biol. Rev..

[141] Sah S.K., Reddy K.R., Li J. (2016). Abscisic acid and abiotic stress tolerance in crop plants. Front. Plant Sci..

[142] Abdelaal K.A., El-shawy E.A., Mohamed Hafez Y., Abdel-dayem S.M.A., Chidya R.C.G., Saneoka H., Sabagh A. (2020). EL Nano-Silver and non-traditional compounds mitigate the adverse effects of net blotch disease of barley in correlation with up-regulation of antioxidant enzymes. Pakistan J. Bot..

[143] Yang L., Liu S., Lin R. (2020). The role of light in regulating seed dormancy and germination. J. Integr. Plant Biol..

[144] Lee J., Kang M.H., Kim J.Y., Lim P.O. (2021). The Role of Light and Circadian Clock in Regulation of Leaf Senescence. Front. Plant Sci..

[145] Mullineaux P.M., Exposito-Rodriguez M., Laissue P.P., Smirnoff N. (2018). ROS-dependent signalling pathways in plants and algae exposed to high light: Comparisons with other eukaryotes. Free Radic. Biol. Med..

[146] Roeber V.M., Bajaj I., Rohde M., Schmülling T., Cortleven A. (2020). Light acts as a stressor and influences abiotic and biotic stress responses in plants. Plant Cell Environ..

[147] Maai E., Nishimura K., Takisawa R., Nakazaki T. (2020). Light stress-induced chloroplast movement and midday depression of photosynthesis in sorghum leaves. Plant Prod. Sci..

[148] Zandalinas S.I., Fritschi F.B., Mittler R. (2021). Global Warming, Climate Change, and Environmental Pollution: Recipe for a Multifactorial Stress Combination Disaster. Trends Plant Sci..

[149] Griebel T., Zeier J. (2008). Light regulation and daytime dependency of inducible plant defenses in *arabidopsis*: Phytochrome signaling controls systemic acquired resistance rather than local defense. Plant Physiol..

[150] Vannier N., Agler M., Hacquard S. (2019). Microbiota-mediated disease resistance in plants. PLoS Pathog..

[151] Trotta A., Rahikainen M., Konert G., Finazzi G., Kangasjärvi S. (2014). Signalling crosstalk in light stress and immune reactions in plants. Philos. Trans. R. Soc. B.

[152] De Vallavieille-Pope C., Huber L., Leconte M., Bethenod O. (2002). Preinoculation effects of light quantity on infection efficiency of *Puccinia striiformis* and *P. triticina* on wheat seedlings. Phytopathology.

[153] Makepeace J.C., Havis N.D., Burke J.I., Oxley S.J.P., Brown J.K.M. (2008). A method of inoculating barley seedlings with *Ramularia collo-cygni*. Plant Pathol..

[154] Li Z., Wakao S., Fischer B., Niyogi K. (2009). Sensing and responding to excess light. Annu. Rev. Plant Biol..

[155] Heiser I., Heß M., Schmidtke K.-U., Vogler U., Miethbauer S., Liebermann B. (2004). Fatty acid peroxidation by rubellin B, C and D, phytotoxins produced by *Ramularia collo-cygni* (Sutton et Waller). Physiol. Mol. Plant Pathol..

[156] Dussart F., Douglas R., Sjökvist E., Hoebe P.N., Spoel S.H., McGrann G.R.D. (2018). Genome-Based Discovery of Polyketide-Derived Secondary Metabolism Pathways in the Barley Pathogen *Ramularia collo-cygni*. Mol. Plant-Microbe Interact..

[157] Dussart F., Jakubczyk D. (2022). Biosynthesis of Rubellins in *Ramularia collo-cygni*—Genetic Basis and Pathway Proposition. Int. J. Mol. Sci..

[158] Daamen R., Stol W. (1992). Surveys of cereal diseases and pests in the Netherlands. 5. Occurrence of *Septoria* spp. in winter wheat. Neth. J. Plant Pathol..

[159] Arraiano L.S., Brading P.A., Brown J.K.M. (2001). A detached seedling leaf technique to study resistance to *Mycosphaerella graminicola* (anamorph *Septoria tritici*) in wheat. Plant Pathol..

[160] (2005). Mycosphaerella pini. EPPO Bull..

[161] Bradshaw R.E. (2004). Dothistroma (red-band) needle blight of pines and the dothistromin toxin: A review. For. Pathol..

[162] Kabir M.S., Ganley R.J., Bradshaw R.E. (2015). Dothistromin toxin is a virulence factor in dothistroma needle blight of pines. Plant Pathol..

[163] Busogoro J.P., Etame J., Lognay G., Messiaen J., Van Cutsem P., Lepoivre P. (2004). Analysis of the mechanisms of action of *Mycosphaerella fijiensis* toxins during the development of black leaf streak disease. Banana Improvement: Cellular, Molecular Biology, and Induced Mutations. Proceedings of A Meeting Held in Leuven, Belgium, 24–28 September 2001.

[164] Noar R.D., Thomas E., Daub M.E. (2022). Genetic Characteristics and Metabolic Interactions between *Pseudocercospora fijiensis* and Banana: Progress toward Controlling Black Sigatoka. Plants.

[165] Hartman P.E., Suzuki C.K., Stack M.E. (1989). Photodynamic production of superoxide in vitro by altertoxins in the presence of reducing agents. Appl. Environ. Microbiol..

[166] Daub M.E., Ehrenshaft M. (2000). The photoactivated *Cercospora* toxin cercosporin: Contributions to plant disease and fundamental biology. Annu. Rev. Phytopathol..

[167] Yoshihara T., Shimanuki T., Araki T., Sakamura S. (1975). Phleichrome; a new phytotoxic compound produced by *Cladosporium phlei*. Agric. Biol. Chem..

[168] Daub M.E., Herrero S., Chung K.R. (2005). Photoactivated perylenequinone toxins in fungal pathogenesis of plants. FEMS Microbiol. Lett..

[169] Upchurch R.G., Walker D.C., Rollins J.A., Ehrenshaft M., Daub M.E. (1991). Mutants of *Cercospora kikuchii* altered in cercosporin synthesis and pathogenicity. Appl. Environ. Microbiol..

[170] Yamazaki S., Okubo A., Akiyama Y., Fuwa K. (1975). Cercosporin: A novel photodynamic pigment isolated from Cercospora kikuchii. Agric. Biol. Chem..

[171] Daub M., Chung K.-R. (2007). Cercosporin: A photoactivated toxin in plant disease. APSnet Featur..

[172] Sloat L.L., Davis S.J., Gerber J.S., Moore F.C., Ray D.K., West P.C., Mueller N.D. (2020). Climate adaptation by crop migration. Nat. Commun..

